# Non-Small-Cell Lung Cancer-Sensitive Detection of the p.Thr790Met EGFR Alteration by Preamplification before PNA-Mediated PCR Clamping and Pyrosequencing

**DOI:** 10.3390/diagnostics10080527

**Published:** 2020-07-29

**Authors:** Amandine Billaud, Veronique Verriele, Jonathan Dauvé, Louise-Marie Chevalier, Alain Morel

**Affiliations:** 1 Université d’Angers, Inserm, CRCINA, F-49000 Angers, France; amandinebillaud@hotmail.fr (A.B.); louisemarie.chevalier@ico.unicancer.fr (L.-M.C.); 2 Institut de Cancérologie de l’Ouest Nantes-Angers, 49000 Angers, France; Veronique.Verriele@ico.unicancer.fr (V.V.); Jonathan.Dauve@ico.unicancer.fr (J.D.)

**Keywords:** peptide nucleic acid, PNA, preamplification, non-small-cell lung cancer, NSCLC, EGFR, p.Thr790Met, T790M, biomarkers, detection sensitivity

## Abstract

Targeted therapies and, more precisely, *EGFR* tyrosine kinase inhibitors (TKIs) have been a major improvement in the therapeutic management of *EGFR*-mutated non-small-cell lung cancers (NSCLCs). Earlier administration of these TKIs throughout tumor progression is imperative to improve patient outcomes. Consequently, studies have focused on refining the characterization of biomarkers, especially concerning the resistance mutation p.Thr790Met of *EGFR*. Herein, we developed peptide nucleic acid (PNA)-mediated PCR clamping followed by pyrosequencing, favoring enrichment of the mutated fraction. A preamplification step was first added to increase the amplifiable DNA fraction. Throughout the application of our method on DNA extracted from FFPE samples of 46 patients with NSCLC who had relapsed under first-generation *EGFR* TKI, we evaluated a sensitivity of 93.3% and a specificity of 100%. All 19 patients who were positive for the p.Thr790Met mutation with NGS were also found to be positive with our protocol. The only discordant case was a sample with no mutation detected with NGS, but which was positive with PNA. This protocol allows for the detection of the p.Thr790Met mutation with a sensitivity of 0.5% which will permit earlier detection and an improvement of therapeutic management.

## 1. Introduction

As a leading cause of cancer-related death worldwide (18.6%) [1], lung cancers are divided into two classes: small-cell lung cancer (SCLC) and non-small-cell lung cancer (NSCLC), which is the most common type (approximately 85%) [2]. Direct exposure to carcinogens through airways and important renewal of the pulmonary epithelium make NSCLC the second most heterogeneous cancer with a major diversity of oncogenic drivers [3]. Despite the predominance of *KRAS* mutations (32%) in those tumors, *EGFR* alterations represent 12% of cases [4,5], affecting mainly the tyrosine kinase domain of the receptor [6], which becomes constitutively active [7,8]. The p.Leu858Arg substitution (40%) and the deletion of exon 19 (45%) are, therefore, the most frequent alterations of *EGFR* [6]. These molecular characterizations led to the appearance of new targeted therapies. The first generation of *EGFR* tyrosine kinase inhibitors (TKIs), gefitinib and erlotinib, improved the 12 month progression-free survival rate, from 6.7% with chemotherapy to 24.9% with gefitinib and from 10% to 40% with erlotinib [9,10,11]. However, after 9 to 12 months of continuous treatment, acquired resistance appears mainly due to a second mutation in *EGFR* that changes the oncogenic addiction [12,13]. The diversity of these mechanisms of resistance is linked to the major tumor heterogeneity of lung cancers [14]. However, the p.Thr790Met alteration of *EGFR* is responsible for more than 50% of acquired resistance to first-generation TKIs, increasing the receptor affinity for ATP and competitively inhibiting TKIs [15]. Targeting this mutation specifically and covalently and bypassing the hemato-encephalic barrier, a third-generation TKI (osimertinib) improved progression-free survival from 4.4 months to 10.1 months compared to platinum therapy plus pemetrexed [16,17]. Consequently, the detection of this alteration has become crucial for its administration.

Following a relapse with first-generation *EGFR* TKIs, a second biopsy is not always possible. Moreover, this invasive technique represents only a snapshot of the disease, without ideas of tumor heterogeneity and evolution in space and time [14,18]. A constant follow up is thus a necessity to detect disease progression earlier and adapt treatment. Consequently, analysis procedures for circulating tumor DNA (ctDNA) and liquid biopsies are developing, resulting in findings of tumor heterogeneity, progression and eventually residual disease [19,20,21]. Concordance between ctDNA and tumor genotype has already been demonstrated, promoting ctDNA as an effective biomarker [22,23]. The quantity of ctDNA detected in blood is a function of tumor stage, localization and oncogene driver and is also associated with patient prognosis [24]. ctDNA can also be detected in non-blood body fluids such as urine, cerebrospinal fluid and pleural liquid [25]. Increasing the sensitivity of mutation detection is a challenge when faced with small concentrations of degraded tumoral DNA (mean of 166 bp) [26] and dilutions in wild-type DNA (mutant allele < 1% of DNA) [27]. Several methods have been used in recent years, including droplet digital PCR, real-time PCR, PNA-LNA PCR clamping and next-generation sequencing (NGS) [28,29,30].

Peptide nucleic acid (PNA) is an artificial DNA with a sugar phosphate backbone that is replaced by pseudopeptide (N-(2-aminoethyl) glycine units), allowing it to bind DNA with higher affinity [31] and to resist exonuclease activities. Included in PCR, PNA hybridizes with the wild-type sequence, blocking the action of the polymerase and, thereby, its amplification. Mutated DNA is preferentially amplified causing enrichment and increasing its detection sensitivity [32]. Herein, we started with a preamplification step increasing the amplifiable fraction before PNA-mediated PCR clamping followed by pyrosequencing. Because of sample scarcity, we proved the efficiency and sensitivity of our protocol by analyzing DNA from somatic tissue.

## 2. Materials and Methods

### 2.1. Patient Samples and DNA Extraction

Tumor specimens were obtained from 46 patients diagnosed with NSCLC between years 2015 and 2018 from the Institut de Cancérologie de l’Ouest (ICO). For all of them, the p.Thr790Met mutation in *EGFR* was assessed by NGS using a S5 apparatus (ThermoFisher) or pyrosequencing and 19 results were positive. The included patients were 23 men and 23 women from ages 50.8 to 87.8. This retrospective study was approved by the local ethics committee of Angers medical university on April 2020 under the reference number 2020-36. This study was conducted on formalin-fixed paraffin-embedded (FFPE) tissue samples (6 h for solid biopsies and 24 h for surgical samples). Hematoxylin and eosin (H&E)-stained slides were used to determine tumor cell content by area. All genomic DNA was extracted from 10 µm sections from FFPE blocks using the NucleoSpin DNA FFPE XS Kit (Macherey Nagel, Düren, Germany,) and stored at −20 °C.

### 2.2. Next-Generation Sequencing

NGS barcoded library preparations of all samples were carried out with 20 ng of DNA with the Ion AmpliSeq 2.0 Library Kit (Thermo Fisher Scientific, Waltham, MA, USA). A pool of primer pairs for entire *EGFR* tyrosin kinase domain and boundaries, among other oncogenic drivers, were used to generate the sequencing libraries. Each read has a mean size of 102 bases, with a total size of 5.2 kb for the whole panel. Clonal amplification of the libraries was carried out by emulsion PCR using an Ion OneTouch 2 (Thermo Fisher Scientific, Waltham, MA, USA) and the Ion PGM Hi-Q View OT2 Kit (Thermo Fisher Scientific, Waltham, MA, USA) according to the manufacturer’s instructions. The prepared libraries were sequenced on the Ion PGM (Thermo Fisher Scientific) using an Ion PGM Hi-Q View Sequencing Kit (Thermo Fisher Scientific, Waltham, MA, USA). Variants of interest were visualized in Integrative Genomics Viewer (IGV), a high performance visualization tool for interactive exploration of large integrated genomic dataset on standard desktop computers. The minimal cover established to analyze a sample was 300X for each position.

### 2.3. Analysis of the Sensitivity and Specificity of the Detection of the p.Thr790Met Alteration

To assess the specificity and sensitivity of our technique, a p.Thr790Met highly mutated sample (50%) was used as a reference standard (Horizon Discovery, Waterbeach, UK), and different dilutions were made from it to establish three ranges. Wild-type DNA was extracted from peripheral blood nuclear cells (PBNC) of three healthy donors, using the Maxwell16 Blood DNA Purification Kit (Promega, Madison, WI, USA). Dilutions were made to obtain the following percentages of p.Thr790Met mutation: 50, 10, 5, 1, 0.5 and 0.1%. The protocol used on patient DNA was also used on these three ranges, and they were pyrosequenced with or without PNA.

### 2.4. PNA-Mediated PCR Clamping on the Wild-Type EGFR Sequence

PNA sequence targeting the genomic region encompassing the p.Thr790Met alteration of *EGFR* (Table 1) was resuspended in DNase/RNase-free water (Gibco) at 100 mm and stored at −20 °C. This concentration was diluted to 10 mm before use. A first step of preamplification with 12 cycles was used to enhance the amplifiable fraction of the DNA. PCR cycling conditions of the preamplification included therefore a 10 min hold at 94 °C followed by 12 cycles of three temperature steps (30 s at 94 °C, 30 s at 57 °C and 1 min at 72 °C) and a 5 min final extension at 72 °C. Five nanograms of genomic DNA was mixed with Ampli Taq Gold DNA polymerase (5 U/µL, Thermo Fisher Scientific, Waltham, MA, USA), dNTP (10 mm, Invitrogen, Carlsbad, CA, USA), primers (4 µm, sequences in Table 1) and an MgCl2 concentration of 1.5 µm. Then, a second PCR amplification was performed using the same primers and mixes and 10 µL of the preamplification products were added followed by 0.2 µm of the PNA targeting the wild-type sequence. The same reactions were also conducted as control experiments without PNA. PCR cycling conditions included a 10 min hold at 94 °C followed by 35 cycles of three temperature steps (30 s at 94 °C, 30 s at 57 °C and 1 min at 72 °C) and a 5 min final extension at 72 °C. Amplifications and sizes were checked with Qiaxcel (Qiagen) before pyrosequencing.

### 2.5. Pyrosequencing

The genomic region encompassing the p.Thr790Met alteration of *EGFR* was characterized by pyrosequencing with PSQ-96MA (Qiagen). Briefly, 40 µL of the second PCR amplification with or without PNA was bound to streptavidin beads. Then, the beads were washed before being added to the sequencing primer (0.2 µm) (Table 1). The samples were incubated at 80 °C for two minutes. dNTPs were added following the dispensation order (Table 1), and the pyrophosphates resulting from the incorporation of dNTPs were measured. Pyrogram outputs were analyzed using Pyromark Q24 (Qiagen) software to determine the percentages of p.Thr790Met mutation over the wild-type sequence.

### 2.6. Statistics

A linear regression of the mutation frequencies was calculated to evaluate the precision of the dilutions before comparison with the results obtained with PNA. A detection limit was calculated with the formula: detection limit = mean (p.Thr790Met frequency) + 3 * SD (p.Thr790Met frequency), using DNA from three healthy donors. The frequency of p.Thr790Met alteration was searched with or without PNA, six times each, in those samples. Κ analysis was also performed to compare our results to those obtained with the technique of reference, NGS. Statistical analyses were performed with GraphPad Prism analysis Software.

## 3. Results

### 3.1. Determination of the Detection Limit of Our PNA-Mediated PCR Clamping

**Figure 1:**
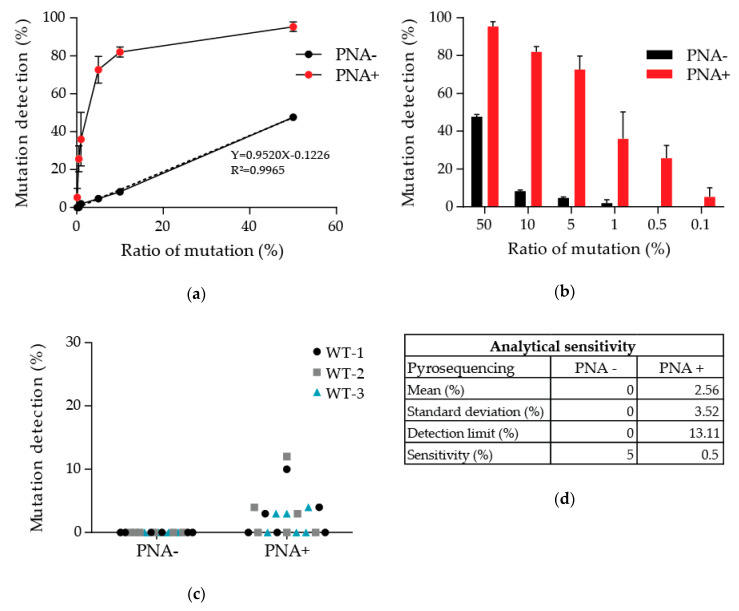
Preamplification followed by PNA-mediated PCR clamping allowed a sensitivity of detection of 0.5% of the p.Thr790Met mutation. (**a**,**b**) Pyrosequencing results of the three ranges with different percentages of the p.Thr790Met mutation of *EGFR,* with or without PNA, after preamplification. (**c**,**d**) DNA from three healthy donor analyses (six times each) was used to evaluate the analytical sensitivity and detection limit.

**Figure A1:**
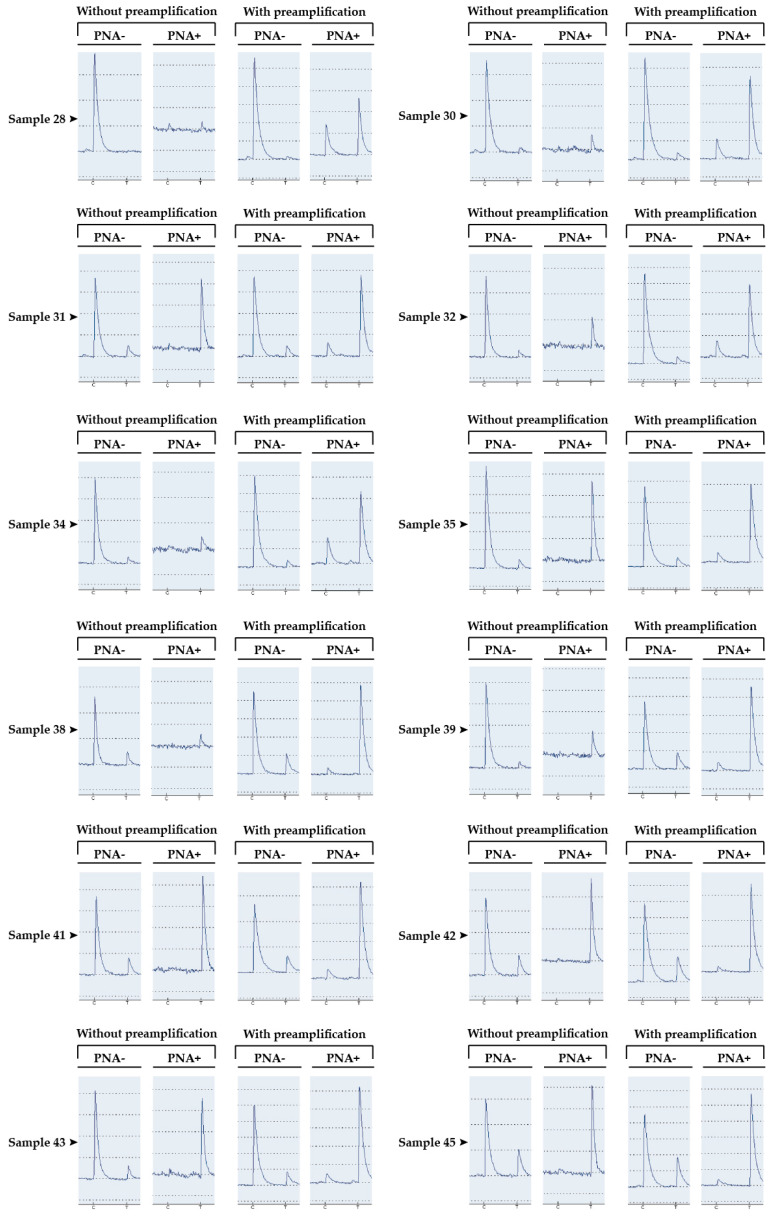
Pyrograms obtained analyzing *EGFR* p.Thr790Met mutated samples with or without PNA and after, or not, a preamplification followed by pyrosequencing.

The sensitivity of our PNA-mediated PCR clamping method was assessed using a range with different percentages, from 50 to 0.1%, of the p.Thr790Met alteration of *EGFR.* Three ranges were realized by diluting an *EGFR* p.Thr790Met reference standard, 50% mutated, in wild-type DNA extracted from lymphocytes of healthy donors to avoid cancer cell aneuploidy, which would perturb the precision of our dilutions. The linearity of our ranges was first verified without PNA, and an R² of 0.9965 was obtained (Figure 1a). PNAs were added to the PCR mix in parallel, and the same protocol was followed to evaluate the p.Thr790Met frequencies at the different points of the ranges. Where PNAs were added, mutation frequencies tended to saturate where there were over five to 10% in the original sample (Figure 1a,b). The different points of the ranges with or without PNA were compared to validate the enrichment of the p.Thr790Met mutation in the samples. Without PNA, the frequencies of the p.Thr790Met alteration in mutated samples are similar whether or not a preamplification had been performed (Table 2). However, pyrogram signals are better after preamplification (Appendix A Figure A1). This is even clearer where PNA had been added. The preamplification step enhanced the fraction of amplifiable DNA allowing the detection of signals, which were otherwise drown in background noise.

**Figure A2:**
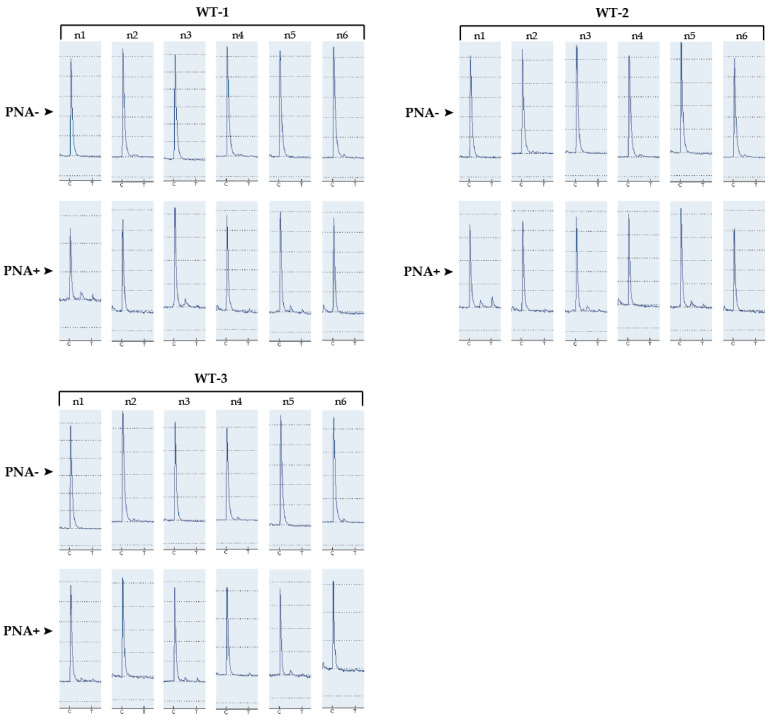
Pyrograms obtained analyzing the *EGFR* p.Thr790Met alteration in DNA extracted from three healthy donors, with or without PNA.

The detection limit of our method was then evaluated. Wild-type DNA extracted from three healthy donors was tested several times with or without PNA (Figure 1c,d). Due to the prior preamplification step, background noise was present with PNAs (Appendix A Figure A2). A limit of detection of 13.11% was consequently calculated (limit = mean + 3*standard deviation) based on the frequencies of the p.Thr790Met alteration detected, which is mainly background noise (Appendix A Figure A2 WT-1 n1 and WT-2 n1 for the highest frequencies). The *EGFR* p.Thr790Met reference standard also allowed the determination of the analytical sensitivity of PNA-mediated PCR clamping followed by pyrosequencing. Contrary to classical pyrosequencing, the detection limit of which remains 5%, the addition of PNA in the PCR mix enabled the detection of smaller percentages up to 0.5%.

### 3.2. Detection of the p.Thr790Met Alteration of EGFR in Patients’ Somatic DNA

**Figure 2:**
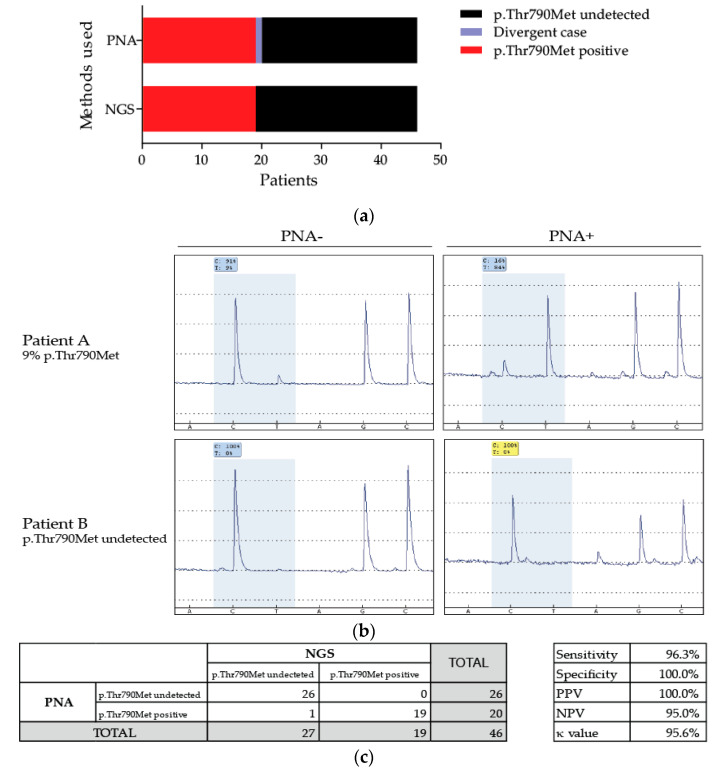
Comparison of the characterization of the p.Thr790Met mutation with NGS and our PNA-mediated method in patients’ somatic DNA. (**a**) Representation of the results obtained after pyrosequencing analysis with PNA compared to those obtained with NGS for the 46 patients. (**b**) Pyrograms of two patients, one with the p.Thr790Met mutation, with or without PNA. (**c**) Analysis of the results to assess the sensitivity and specificity of our method compared to the method of reference, NGS.

Because of ctDNA sample scarcity and poor DNA concentration and quality, we applied our method to the analysis of DNA extracted from solid tumors. Somatic DNA extracted from 46 patients, suffering from NSCLC was first tested using NGS analysis for the presence of the p.Thr790Met alteration. This resistance alteration was detected in 19 samples (Figure 2a). Preamplification followed by PCR with and without PNA and pyrosequencing was performed for each sample. Herein, the pyrograms obtained for mutated and wild-type samples with and without PNA are represented (Figure 2b). An enrichment of the mutation frequencies was noticed for all mutated samples, increasing from two to 26% with NGS to over 28% with PNA (Table 3). All the results were compared to those obtained with NGS (Table 3 and Figure 2a). Seven samples that were found wild type had a few percentages with PNA but were below the calculated limit of detection, so they were considered wild type. Only one patient had discordant results. He was wild type with NGS but was found positive with our PNA-mediated PCR method. Consequently, the comparative analysis of all results allowed us to determine a sensitivity of 96.3% and a specificity of 100% with our protocol with a κ value of 95.6% (Figure 2c). The positive predictive value (PPV) was 100% and the negative predictive (NPV) value was 95%.

### 3.3. Patient with Discordant Results

**Figure 3:**
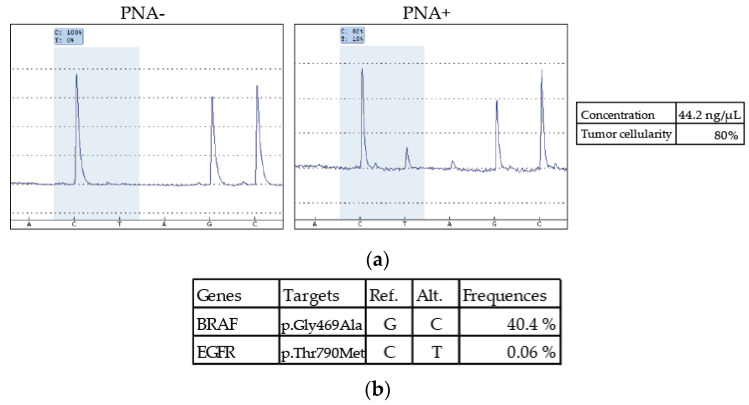
Results of the discordant case that was mutated with the PNA method. (**a**) Pyrograms obtained with and without PNA (*n* = 2). (**b**) Results obtained for the same patient with NGS.

The somatic DNA from the discordant sample presented a tumor cellularity of 80% and a concentration of 44.2 ng/µL. No mutation was detected without PNA, but its addition allowed the appearance of an 18% peak at the p.Thr790Met alteration localization, so above the limit of detection calculated earlier (Figure 3a). This patient was a 51-year-old woman who had breast cancer in 2015 treated by surgery and hormonotherapy and was diagnosed with advanced pulmonary adenocarcinoma in 2017. NGS was performed and a *BRAF* mutation was detected with a frequency of 40.4% (Figure 3b). Following our analysis with PNA and pyrosequencing, we searched for the p.Thr790Met mutation of *EGFR* in the NGS results. Indeed, only two reads were detected, giving a frequency of 0.06% so far under the threshold of NGS.

## 4. Discussion

Over half of the acquired resistance to first-generation *EGFR* tyrosine kinase inhibitors in NSCLC is due to the appearance of the p.Thr790Met variant of *EGFR*. Currently, the acquisition of this mutation is no longer such a poor prognosis thanks to a third-generation *EGFR* TKI called osimertinib. Consequently, there is a need for a highly sensitive method of detection for this variant, enabling an earlier adaptation of treatment. Herein, we described an improvement of the PNA-mediated PCR clamping method preceding pyrosequencing. Indeed, a first step of preamplification, without PNA enhanced the fraction of amplifiable DNA (Table 2 and Appendix A Figure A1). We therefore were able to demonstrate an analytical sensitivity of 0.5% with a detection limit of 13.11% due to background noise (Figure 1c,d and Appendix A Figure A2). The second amplification with PNA allowed an enrichment of the mutated fraction of the sample and tended to saturate where there was more than 10% mutation in the original sample (Figure 1a,b). Hence, our method favors an easier and inexpensive detection of small percentages and is qualitative rather than quantitative.

To evaluate the sensitivity and specificity of our method, we analyzed 46 DNA samples extracted from solid tumors from patients who relapsed after the appearance of acquired resistance to first-generation *EGFR* TKIs. NGS, our method of reference, determined that 19 of them had this resistance alteration. They were all also positive after utilization of PNA (Figure 2a and Table 3). Only one discordant sample, which was wild type with NGS, was positive for the p.Thr790Met alteration with PNA (Table 3, Figure 2a and Figure 3a). These results allowed the calculation of a κ-value of 0.956 with a sensitivity of 96.3% and a specificity of 100%, resulting in very strong agreement [33] (Figure 2c). Moreover, the analytical sensitivity was greatly improved (over 0.5%) but to the detriment of the statistical sensitivity for such small percentages. However, when we closely analyzed the NGS results from the discordant case, which had a frequency of 18% of the p.Thr790Met variant with PNA (Figure 3a), two reads indeed carried this alteration, giving a frequency of 0.06%, below its threshold of 2% (Figure 3b). This discordant case could truly be mutated, thereby favoring a good statistical sensitivity of our method.

Currently, the third-generation *EGFR* TKI (osimertinib) is now accessible as a first-line treatment where an activating mutation of *EGFR* is characterized in a patient with NSCLC. A longer progression-free survival was shown compared to first-line *EGFR* TKI, and the p.Thr790Met alteration is no longer a factor of resistance to that treatment [34]. However, after a few months of continuing treatment, acquired resistance can also appear, partially related to the p.Cys797Ser mutation of *EGFR* [35,36]. The method developed herein could be perfectly applied to the analysis of this mutation or to other punctual alterations, such as the hotspots of *PIK3CA* or the p.Val600Glu alteration of *BRAF*. Despite the low cost and rapidity of analysis of pyrosequencing, NGS remains the method of reference, allowing simultaneous characterization of many hotspots of different genes. However, the test presented here could be used to confirm those results especially where small percentages are detected. This method could also provide an answer to a persistent question concerning the mechanisms responsible for acquired resistance: is it the selection of a pre-existent resistant clone, its apparition under treatment pressure or a combination of both hypotheses? First studies were more in favor of treatment pressure, but recent analyses with more sensitive techniques [37,38], in which our method could participate, dispute this assertion.

Finally, the developed protocol could also be used to address the problems of the study of circulating tumor DNA. These samples are fragmented, weakly concentrated and drowned in constitutive blood DNA. The first preamplification step allowing enrichment of amplifiable DNA, our PNA-mediated PCR clamping, could thereby be a solution. It could then be used to follow treatment efficiency and the disappearance of the targeted activating mutation. Furthermore, it can be used to evaluate eventual residual disease or to detect a putative acquired resistance mechanism earlier. All possibilities in molecular detection are unified towards a common goal: early adaptation of even more personalized treatments in the field of oncology.

## Figures and Tables

**Table 1:** Primers and peptide nucleic acid sequences.

Names	Sequences
PCR forward primer	5′-GCA TCT GCC TCA CCT CCA A-3′
PCR reverse primer	5′-biotin-CGA TCT GCA CAC ACC AGT TG-3′
PNA sequence	NH2-CTCATCACGCAGCTCA-COOH [32]
Sequencing primer (pyrosequencing)	CCG TGC AGC TCA TCA
Dispensation order (pyrosequencing)	ACTAGCAGC

**Table 2:** Evaluation of the p.Thr790Met frequency of *EGFR* by pyrosequencing in mutated samples after a preamplification step, with and without PNA.

Samples	Without Preamplification		With Preamplification	
	PNA− (%)	PNA+ (%)	PNA− (%)	PNA+ (%)
Sample 28	0	NA	2	28
Sample 30	5	NA	5	59
Sample 31	12	100	10	68
Sample 32	8	NA	7	72
Sample 34	7	NA	9	84
Sample 35	8	100	9	84
Sample 38	16	NA	22	88
Sample 39	22	100	20	90
Sample 41	18	100	20	91
Sample 42	21	100	23	91
Sample 43	13	92	11	92
Sample 45	26	100	28	93

**Table 3:** Characterization of the p.Thr790Met of *EGFR* in the 46 samples by next-generation sequencing or preamplification and pyrosequencing with or without PNA.

Samples	Ages	Sexes	[DNA] (ng/µL)	Cellularity	NGS Results	PNA-Mediated PCR		Concordance Results
						PNA−	PNA+	
Sample 1	50.8	M	10.7	30	WT	0	0	OK-WT
Sample 2	53.7	W	14.2	40	WT	0	0	OK-WT
Sample 3	62.1	M	25.9	20	WT	0	0	OK-WT
Sample 4	55.5	W	10.2	20	WT	0	0	OK-WT
Sample 5	62.7	M	13.3	30	WT	0	0	OK-WT
Sample 6	73.4	M	39	10	WT	0	0	OK-WT
Sample 7	64.2	M	6.4	20	WT	0	0	OK-WT
Sample 8	67.2	M	6.6	40	WT	0	0	OK-WT
Sample 9	51.4	W	11.6	40	WT	0	0	OK-WT
Sample 10	81.6	M	11.4	20	WT	0	0	OK-WT
Sample 11	56.6	M	52.8	70	WT	0	0	OK-WT
Sample 12	83.6	W	1.4	10	WT	0	0	OK-WT
Sample 13	56	M	15.6	30	WT	0	0	OK-WT
Sample 14	52.7	M	7.2	30	WT	0	0	OK-WT
Sample 15	67.6	M	10.7	30	WT	0	0	OK-WT
Sample 16	59	W	18.2	80	WT	0	0	OK-WT
Sample 17	69.7	M	29.7	60	WT	0	0	OK-WT
Sample 18	56.2	M	5.7	60	WT	0	0	OK-WT
Sample 19	62.4	W	2.9	5	WT	0	0	OK-WT
Sample 20	63.2	W	9.5	70	WT	0	5	L-WT
Sample 21	71.4	M	24.5	40	WT	0	5	L-WT
Sample 22	82	M	9.5	10	WT	0	5	L-WT
Sample 23	61.1	M	16.1	60	WT	0	6	L-WT
Sample 24	83	W	5.5	20	WT	0	6	L-WT
Sample 25	NA	W	NA	NA	WT	0	6	L-WT
Sample 26	62.3	M	24.8	30	WT	0	8	L-WT
Sample 27	51.4	W	44.2	40	WT	0	18	Discordant
Sample 28	86.7	W	27.3	5	T790M	2	28	OK-Mutated
Sample 29	NA	M	7.4	NA	T790M (1.3%)	2	35	OK-Mutated
Sample 30	76.4	W	10	40	T790M (3.5%)	5	59	OK-Mutated
Sample 31	74.6	M	12.4	4	T790M	10	68	OK-Mutated
Sample 32	78.2	W	2.2	10	T790M	7	72	OK-Mutated
Sample 33	73.7	W	2.1	50	T790M (9%)	11	75	OK-Mutated
Sample 34	67.2	M	4.1	10	T790M	9	84	OK-Mutated
Sample 35	75.6	W	23.7	40	T790M	9	84	OK-Mutated
Sample 36	87.8	W	12.4	20	T790M (12%)	24	87	OK-Mutated
Sample 37	66.4	W	6	80	T790M	26	87	OK-Mutated
Sample 38	64.3	W	2.5	60	T790M	22	88	OK-Mutated
Sample 39	64.8	W	9.4	40	T790M	20	90	OK-Mutated
Sample 40	87.6	W	4.4	50	T790M (15.4%)	17	90	OK-Mutated
Sample 41	65.3	M	30.2	10	T790M	20	91	OK-Mutated
Sample 42	NA	M	6.4	NA	T790M (18.1%)	23	91	OK-Mutated
Sample 43	78.3	W	30.9	70	T790M	11	92	OK-Mutated
Sample 44	77	W	0.6	10	T790M (14.7%)	23	92	OK-Mutated
Sample 45	74	W	6.9	40	T790M	28	93	OK-Mutated
Sample 46	78.5	M	3.8	60	T790M (21.3%)	26	95	OK-Mutated

Concordant results of wild-type patients for this alteration are in green (OK-WT), and mutated patients are in red (OK-Mutated). Patients with a few percentages of this mutation with PNA but below the calculated limit of detection of the method are in yellow (L-WT). The discordant patient is in blue (Discordant). NA: not applicable.

## Abbreviations

TKI	tyrosine kinase inhibitor
NSCLC	non-small-cell lung cancer
PNA	peptide nucleic acid
SCLC	small-cell lung cancer
ctDNA	circulating tumor DNA
NGS	next-generation sequencing
PBNC	peripheral blood nuclear cells
FFPE	formalin-fixed paraffin-embedded
ICO	Institut de Cancérologie de l’Ouest
